# Avoidable Hospitalization Trends From Ambulatory Care-Sensitive Conditions in the Public Health System in México

**DOI:** 10.3389/fpubh.2021.765318

**Published:** 2022-01-21

**Authors:** Ofelia Poblano Verástegui, Laura del Pilar Torres-Arreola, Sergio Flores-Hernández, Armando Nevarez Sida, Pedro J. Saturno Hernández

**Affiliations:** ^1^CIEE National Institute of Public Health, Cuernavaca, Mexico; ^2^Epidemiologic and Health Services Research Unit, Aging Area, CMNSXXI, Mexican Institute of Social Security, México City, Mexico

**Keywords:** trends, ACSC, regionalization, join point regression, avoidable hospitalizations

## Abstract

**Objectives:**

To estimate and identify the variations in rates of Avoidable Hospitalization for Ambulatory Care Sensitive Conditions (AH-ACSC) in public institutions of the Mexican health system during the period 2010–2017.

**Methods:**

Secondary analysis of the hospital discharge database of the Ministry of Health (MoH) from 2010 to 2017. AH for ACSC was calculated by age group and sex per 100,000. Variations per year between institutions were calculated with the extreme quotient (EQ), coefficient of variation (CV) and systematic component of variance (SCV). Adjusted AH rates were calculated by group of causes (acute, chronic and preventable by vaccination). Adjusted AH trend rates were analyzed by Join Point Regression.

**Results:**

For the period 2010–2017, the number of AH for ACSC decreased from 676,705 to 612,897, going from almost 13% to 10.7% of hospital discharges. There is consistency in terms of relative variance magnitude. But, with regards to SCV, the change remained constant, and in a second period of 2015–2017, high variation was observed by SCV ≥ 3. All-cause AH is diminishing in all institutions. AH rates for diabetes are the highest, but like other chronic diseases, there was a decline in the period from 2010 to 2017. The relative reduction varied from 15% for heart failure to 38% for complications from diabetes or hypertension, to 75% for angina.

**Conclusions:**

AH for ACSC is an indirect indicator of quality and access to first-level care. Variations by institutions are observed. This variation in CV and SCV across subsystems and states may be due to inequities in the provision of services. The factors that contribute to the burden of AH for ACSC in the Mexican Health System require detailed analysis.

## Introduction

The efficiency and effectiveness of health systems has been a priority for all countries, and one of the challenges in health policy. This has focused on the need of developing indicators of health system performance, as well as specific indicators of quality of care at the different levels. Avoidable hospitalizations (AH) for ambulatory care-sensitive conditions (ACSC) has been considered an indicator of quality of care at the primary care level. ACSC has been defined as those “conditions where good outpatient care can potentially prevent the need for hospitalization” (1). That is, those diseases sensitive to prevention, diagnosis and treatment on an outpatient basis, which can be adequately and timely attended at the first level of care, such as acute conditions, which could be preventable, if timely access and provision of services is guaranteed on an outpatient basis, and, in the case of certain chronic diseases such as diabetes mellitus and hypertension, surveillance and control at the primary care could prevent complications caused by hospitalization (2).

Weissman (3), from a panel of experts, defined a list of 12 ACSC, which has been used by many authors, Gusmano (4) among others, whose study compared the differences in AH for ACSC in large cities, using it as an indicator of equity and efficiency of the health system, and as an indicator of access to primary care (4). In 2009, Purdy (5) conducted a study with the aim of exploring the different ACSC codes related to potentially avoidable hospitalizations and proposed a list of 19 codes. Since then, various authors have used these proposals as a starting point to define by consensus the most appropriate codes according to their context, and some have considered the importance of defining categories that group codes, considering whether it is an acute, chronic or preventable condition (6, 7).

ACSC hospitalization rates vary by geographic area and by population group, suggesting that characteristics related to availability of and access to primary care services and timely outpatient care are determining factors (6). AH for ACSC has been considered as a proxy indicator of the performance of primary care in many countries (8–10). Primary care (the first level of care) is the initial point of contact with the health care system. When care is continuous, comprehensive and coordinated, it should reduce a large number of events and chronic disease complications, and promote better use of resources (11).

The results of some studies show that high rates of AH suggest a great disparity in access to primary care services (12). The results of studies performed in the US have evidenced that high rates of AH are greater in a population with limited medical coverage, such as the case of the Hispanic population with low socioeconomic level, and in areas where the providers of primary care are scarce (8). Other studies have shown that the socioeconomic level is a determining factor of AH for ACSC (13).

In the last two decades, the behavior of AH for ACSC has varied by period and public health system institution in Mexico.

The health system in Mexico is characterized by being a system of government with vertical public institutions, which has been generated through agreements through a corporatist policy, with little or no participation of citizens, patients or consumers of services.

The National Health System (NHS) in Mexico is a segmented system, with different health subsystems that historically have involved different social security institutions at the federal and state levels, as well as a subsystem of health protection, formerly called System for Social Protection in Health (SPSS, by its acronym in Spanish) colloquially known as Seguro Popular which between 2013 and 2018 was responsible for providing health coverage to the population not covered by any of the social security institutions. In addition, the NHS considers within its sector a private health care subsystem, where the payment is mainly out-of-pocket or by insurance companies.

The social security public institutions in the NHS are:

1. The Mexican Institute of Social Security (IMSS, by its acronym in Spanish), responsible for the provision of services to 32% of workers in the private formal sector and their families.
2. The Institute for Social Security and Services for State Workers (ISSSTE, by its acronym in Spanish), whose coverage is 7.4% of total federal government workers.
3. Petróleos Mexicanos (PEMEX), the Secretariat of Defense (SEDENA, by its acronym in Spanish) and the Secretariat of the Navy (SEMAR, by its acronym in Spanish) cover ~2% of the total population with social security.

Seguro Popular, until 2018, was responsible for providing services to the population without social security. It reached a coverage of >57.2 million people corresponding to 43.5% of the whole population (14–16). Currently, these services are covered by the recently created National Institute of Health for Welfare (INSABI, by its acronym in Spanish) which operates under the same scheme as the SPSS.

Private medical services account for 44% of the demand for outpatient services and 21% of hospital admissions nationwide (17).

For 2001–2009, IMSS showed a decrease in the age-adjusted AH from 87.5 to 72.6 per 10,000 people and 56% of the causes were diabetes mellitus, gastroenteritis, respiratory diseases, prenatal care and delivery, and urinary tract infections (18). For 2001–2011, there was an increase in AH for ACSC, according to a study by Lugo-Palacios (19), who analyzed hospital discharges at the state and jurisdictional level in 248 public hospitals of the MoH. More recently, it has only been reported that the volume of AH from diabetes mellitus (DM) is ACSC, as well as the burden of the disease due to secondary complications to DM, as a determinant of AH rates (20, 21).

AH for ACSC can be preventable by receiving timely and safe care, thus avoiding complications that lead patients to unnecessary hospitalization. This reality is an element that should be emphasized to health professionals at the first level of care, since in the new reality that the COVID-19 pandemic has forced us to face, the primary care has a preponderant role in their reduction.

The objective of this report is to: (a) estimate the AH rates for ACSC in public institutions of the Mexican health system during the period 2010–2017; (b) identify the variations in the main public institutions of the health sector in Mexico during the period 2010–2017.

## Methods

A secondary analysis of databases on ACSC hospitalizations in Mexico from 2010 to 2017 was performed. The main source of information was the Automated System of Hospital Discharges (SAEH, by its acronym in Spanish) of the three most important institutions in Mexico, regarding coverage of the health system: IMSS, ISSSTE, and SSA.

### Standardization by Age and Sex

Crude rates for avoidable hospitalizations were calculated by age groups and sex per 100,000; by year, institution, state and cause; the numerator was the corresponding preventable discharges, calculated with the sectoral hospital discharges base from the MoH (22).

As denominator, the population at mid-year for Mexico and by state was used, estimated by CONAPO (23) for the years 2010–2017. In the case of rates by institution, we used the entitled population of the ISSSTE and the populations not covered by social security reported in the MoH dynamic cubes (24) and in the IMSS, the enrolled insured population (Mexican Institute of Social Security, accessed in 2021) (25).

Regarding ACSC as causes of avoidable hospitalizations, we took as a starting point the classification of Purdy 2009 (5), which included 19 categories, adding two additional categories that apply to their context in Mexico based on previous studies (17, 26, 27). These 21 categories of ACSC were grouped into acute, chronic and preventable by vaccination, using the classification proposed by Page (7). The categories and ICD 10 codes associated are shown in Table 1.

**Table 1:** ACSC categories, ICD 10 codes.

**Category**	**ICD10 codes (Purdy)**	**ICD 10 codes (26); (18); (27)**
**Vaccine preventable**		
Influenza and pneumonia (I&P)	J10, J11, J13, J14, J15.3, J15.4, J15.7, J15.9, J16.8, J18.1, J18, J189, J120, J121, J122, J128, J129, J160, A481, A70x	J13-J14, J15.3–J15.4, J15.8–J15.9, J18.1
Other vaccine-preventable diseases (OVPD)	A35, A36, A37, A80, B05, B06, B16.1, B16.9, B18.0, B18.1, B26, G00.0, M01.4	A33–37, A95, B16, B05–B06, B26, G00.0, A17.0, A19
**Chronic**		
Diabetes complications (DC)	E10.0–E10.8, E11.0–E11.8, E12.0–E12.8, E13.0–E13.8, E14.0–E14.8, E139, E149	E10–E14
Nutritional deficiency (NUT)	E40, E41, E42, E43, E55.0, E64.3	E40–E46, E50–E64
Iron-deficiency anemia (AN)	D50.1, D50.8, D50.9, D460, D461, D463, D464, D510–D513, D518, D520, D521, D528, D529, D531, D571, D580, D581, D590–D592, D599, D601, D608, D609, D610, D611, D640–D644, D648	D50
Hypertension (HYPERT)	I10, I11.9	I10–I11
Congestive heart failure (HEART)	I11.0, I50, J81, I130, I255	I50, J81
Angina (ANG)	I20, I24.0, I24.8, I24.9, I25, R072, R073, R074, Z034, Z035	I20
Chronic obstructive pulmonary disease (COPD)	J20, J41, J42, J43, J47, J44, J40X	
Asthma (ASTH)	J45, J46	J45–J46
**Acute**		
Dehydration and gastroenteritis (GASTRO)	E86, K52.2, K52.8, K52.9, A020, A04, A059, A072, A080, A081, A083, A084, A085, A09, K520, K521	E86, A00–A09
Convulsions and epilepsy (EPILEP)	G40, G41, R560, O15, G253, R568	G40–G41
Ear, nose and throat infections (EN&T INFEC)	H66, H67, J02, J03, J040, J06, J31.2	J20, J21, J40–J44, J47
Dental conditions (DENTAL)	A69.0, K02, K03, K04, K05, K06, K08, K09.8, K09.9, K12, K13	
Perforated or bleeding ulcer (ULCER)	K25.0–K25.2, K25.4–K25.6, K26.0–K26.2, K26.4–K26.6, K27.0–K27.2, K27.4–K27.6, K28.0–K28.2, K28.4–K28.6, K920, K921, K922, K20x, K210, K219, K221, K226	K25–K28, K92.0, K92.1, K92.2
Pyelonephritis (PYELO)	N10, N11, N12, N13.6, N300, N390, N159, N308, N309	N10–N12, N30, N34, N39.0
Pelvic inflammatory disease (PELVIC)	N70, N73, N74	N70–N73, N75–N76
Cellulitis (CELL)	L03, L04, L08.0, L08.8, L08.9, L88, L98.0, I891, L010, L011, L020–L024, L028, L029	A46, L01–L04, L08
Gangrene (GAN)	R02	
Avoidable Conditions (AC)		A15–A16, A18, A17.1–A17.9, I00–I02, A51–A53, B50–B54, B77
Diseases related with the prenatal health care of pregnancy and delivery (DPCPD)		O23, AS0, P35.0

Using direct standardization, adjusted rates were calculated, with 95% confidence interval and standard error, by age group and sex per 100,000 for avoidable hospitalizations, by year, institution, state and cause. In order to take into account the aging process of each of the populations, the population corresponding to the year 2019 was used as the standard population.

Calculation of adjusted rates was performed in the statistical package Stata version 16.0 (Stata, Stata Corp, College Station, TX).

### Quantifying the Variation

The variations per year between institutions were calculated using the extreme quotient (EQ), coefficient of variation (CV) and systematic component of variance (SCV). EQ was calculated by the ratio of 95th percentile between 5th percentile, while the CV is given by the standard deviation between the average rates, and finally the SCV was calculated according to McPherson (28) and, variations >3 in the SCV suggested important differences in clinical practice or in medical criteria:


$$\begin{array}{l} SCV=\left(\frac{\sum_{i}\frac{{\left(ORi-ERi\right)}^{2}}{{ERi}^{2}}-\;\sum_{i}\frac{1}{ERi}}{n-1}\right)x\;100 \end{array}$$

### Join Point Regression

By cause group (acute, chronic, and vaccine-preventable) for each institution, age, and sex, adjusted AH rates were calculated. Trends in adjusted AH rates were analyzed by Joint Point Regression (29) using the Join Point Regression Program (30) version 4.8.0.1.

The years where trend changes occurred, the annual percentage of change (APC), as well as the average annual percentage of change (AAPC) over the entire period (2010–2017) were identified.

Join points were identified using a series of permutation tests, with a significance level of 0.05 using a Monte Carlo method and a natural log-lineal model (31).

## Results

Table 2 shows the crude and age- and sex-standardized rates of AH for ACSC per year and their variation. The number of hospitalizations for ACSC decreased from 676,705 to 612,897, going from almost 13% to 10.7% of hospital admissions. For the standardized rate, there was a change from 638 to 503 per 100,000 habitants.

**Table 2:** Global hospital discharge in Mexico: Variation 2010–2017.

**Year**	**Hospital discharge (A)**	**ACSC (B)**	**%ACSC: (B/A) × 100**	**Crude rate**	**ACSC Age/Sex adjusted rate**	**95% CI adjusted rate**		EQ^*^	CV^*^	SCV^*^
2010	5,207,628	676,705	12.99	594.8	638.8	637.3	640.4	1.57	0.25	0.95
2011	5,412,755	688,426	12.72	596.7	635.8	634.3	637.3	1.53	0.24	0.85
2012	5,536,785	705,059	12.73	602.9	636.9	635.4	638.4	1.49	0.23	0.51
2013	5,554,088	711,093	12.80	600.3	628.8	627.4	630.3	1.52	0.26	2.40
2014	6,276,131	686,516	10.94	572.4	597.1	595.6	598.5	1.63	0.28	2.63
2015	6,260,079	675,191	10.79	556.4	576	574.6	577.3	1.64	0.28	3.18
2016	5,590,447	665,156	11.90	542.0	556.7	555.4	558.0	1.50	0.23	2.98
2017	5,715,854	612,897	10.72	494.1	503.2	502.0	504.5	1.44	0.21	3.47
Total	45,553,767	5,421,043	11.95							

* *CV, Coefficient of variation; RV, Ratio of variation; SCV, Systematic component of variance*.

There appears to be little change between the different measures of variation, and there is consistency in terms of the relative magnitude of variation. But, with respect to SCV, the change remains constant, and in a second period of 2015 to 2017, high variation by SCV ≥ 3 is observed (according to McPherson) (27).

Table 3. By clinical conditions, the evolution of the rates of hospitalizations by ACSC over time was variable. Hospitalizations for diabetes complications were high and, similar to other chronic conditions as they decreased from 2010 to 2017. The relative decrease ranged from 15% for heart failure, 38% for complications from diabetes or hypertension, and up to 75% for angina. There were no changes in the rates for vaccine preventable conditions and increased rates for acute conditions.

**Table 3:** ASCS by cause, age/sex adjusted rate (IC 95%)–Period 2010–2017.

	**2010**	**2011**	**2012**	**2013**	**2014**	**2015**	**2016**	**2017**
Iron-deficiency anemia	3.7	3.8	4.0	3.8	3.8	4.0	4.3	4.0
	(3.6–3.8)	(3.7–3.9)	(3.9–4.1)	(3.7–3.9)	(3.7–3.9)	(3.8–4.1)	(4.2–4.4)	(3.9–4.1)
Angina	40.2	40.0	40.0	38.1	37.2	36.6	34.9	31.5
	(39.8–40.6)	(39.6–40.4)	(39.6–40.3)	(37.7–38.4)	(36.8–37.6)	(36.3–37)	(34.6–35.2)	(31.2–31.8)
Asthma	24.0	22.2	22.6	20.4	22.2	17.6	17.9	13.7
	(23.7–24.3)	(22–22.5)	(22.4–22.9)	(20.1–20.6)	(22–22.5)	(17.4–17.9)	(17.7–18.1)	(13.5–13.9)
Cellulitis	28.1	30.8	31.0	30.4	32.8	33.2	33.5	31.2
	(27.8–28.4)	(30.4–31.1)	(30.7–31.3)	(30–30.7)	(32.5–33.2)	(32.9–33.5)	(33.1–33.8)	(30.9–31.6)
Avoidable conditions	1.2	1.2	1.1	1.1	1.1	1.1	1.0	1.0
	(1.1–1.3)	(1.1–1.2)	(1–1.1)	(1–1.1)	(1–1.2)	(1–1.1)	(1–1.1)	(0.9–1)
Chronic obstructive pulmonary disease	46.2	40.6	38.4	40.4	37.6	35.8	34.9	30.6
	(45.7–46.6)	(40.2–41)	(38.1–38.8)	(40.1–40.8)	(37.2–37.9)	(35.4–36.1)	(34.6–35.3)	(30.3–30.9)
Diabetes complications	111.2	108.2	105.2	100.1	101.5	97.9	88.9	80.3
	(110.5–111.9)	(107.6–108.9)	(104.6–105.8)	(99.5–100.7)	(100.9–102.1)	(97.3–98.5)	(88.4–89.5)	(79.8–80.8)
Dental conditions	6.2	7.0	6.0	6.6	10.7	9.0	5.3	5.7
	(6–6.3)	(6.9–7.2)	(5.9–6.2)	(6.5–6.8)	(10.5–10.9)	(8.8–9.1)	(5.2–5.5)	(5.5–5.8)
Ear, nose and throat infections	17.7	15.8	14.7	15.4	17.4	15.1	13.2	12.9
	(17.5–17.9)	(15.6–16)	(14.5–15)	(15.2–15.7)	(17.2–17.6)	(14.9–15.3)	(13–13.4)	(12.7–13.1)
Convulsions and epilepsy	25.3	25.5	25.9	26.1	27.1	26.7	27.3	24.8
	(25–25.6)	(25.2–25.8)	(25.6–26.2)	(25.8–26.4)	(26.8–27.4)	(26.4–26.9)	(27.1–27.6)	(24.6–25.1)
Gangrene	1.0	1.0	1.0	1.0	1.1	1.0	0.9	0.9
	(0.9–1.1)	(0.9–1.1)	(0.9–1.1)	(0.9–1)	(1–1.1)	(1–1.1)	(0.8–0.9)	(0.8–0.9)
Dehydration and gastroenteritis	56.5	57.0	60.2	57.4	13.8	12.7	12.4	10.0
	(56.1–57)	(56.6–57.4)	(59.7–60.6)	(56.9–57.8)	(13.6–14)	(12.4–12.9)	(12.2–12.6)	(9.8–10.2)
Congestive heart failure	34.1	34.3	33.6	32.2	31.2	31.0	31.4	29.6
	(33.7–34.4)	(33.9–34.7)	(33.2–33.9)	(31.8–32.5)	(30.8–31.5)	(30.7–31.3)	(31.1–31.7)	(29.3–29.9)
Hypertension	37.5	38.7	38.9	36.3	34.6	32.7	31.4	27.1
	(37.1–37.9)	(38.3–39.1)	(38.5–39.3)	(35.9–36.6)	(34.3–34.9)	(32.3–33)	(31.1–31.7)	(26.8–27.4)
Influenza and pneumonia	74.8	74.3	73.4	78.2	78.9	75.7	78.1	73.0
	(74.3–75.3)	(73.8–74.8)	(72.9–73.9)	(77.6–78.7)	(78.4–79.4)	(75.2–76.2)	(77.6–78.6)	(72.5–73.4)
Nutritional deficiency	1.4	1.2	1.2	1.3	1.3	1.2	1.2	0.9
	(1.3–1.5)	(1.2–1.3)	(1.2–1.3)	(1.2–1.4)	(1.2–1.4)	(1.2–1.3)	(1.1–1.3)	(0.9–1)
Other vaccine-preventable diseases	0.9	1.2	1.7	1.4	1.2	1.3	1.0	1.0
	(0.9–1)	(1.2–1.3)	(1.6–1.7)	(1.4–1.5)	(1.1–1.3)	(1.3–1.4)	(1–1.1)	(1–1.1)
Pelvic inflammatory disease	5.6	5.6	5.4	5.3	5.4	5.1	4.8	4.6
	(5.4–5.7)	(5.4–5.7)	(5.3–5.6)	(5.2–5.4)	(5.2–5.5)	(5–5.2)	(4.7–5)	(4.5–4.7)
Prenatal health care	45.1	44.3	46.1	46.1	46.3	45.4	42.7	35.1
	(44.7–45.5)	(43.9–44.7)	(45.7–46.5)	(45.7–46.5)	(46–46.7)	(45–45.8)	(42.4–43.1)	(34.8–35.4)
Pyelonephritis	40.1	43.2	46.4	46.8	51.1	51.8	51.9	47.1
	(39.7–40.5)	(42.8–43.6)	(46–46.8)	(46.4–47.2)	(50.6–51.5)	(51.4–52.2)	(51.5–52.3)	(46.7–47.4)
Perforated or bleending ulcer	38.5	40.0	40.2	40.8	41.1	41.5	39.7	38.6
	(38.2–38.9)	(39.6–40.4)	(39.8–40.6)	(40.4–41.2)	(40.7–41.5)	(41.1–41.9)	(39.4–40.1)	(38.2–38.9)

**Figure 1:**
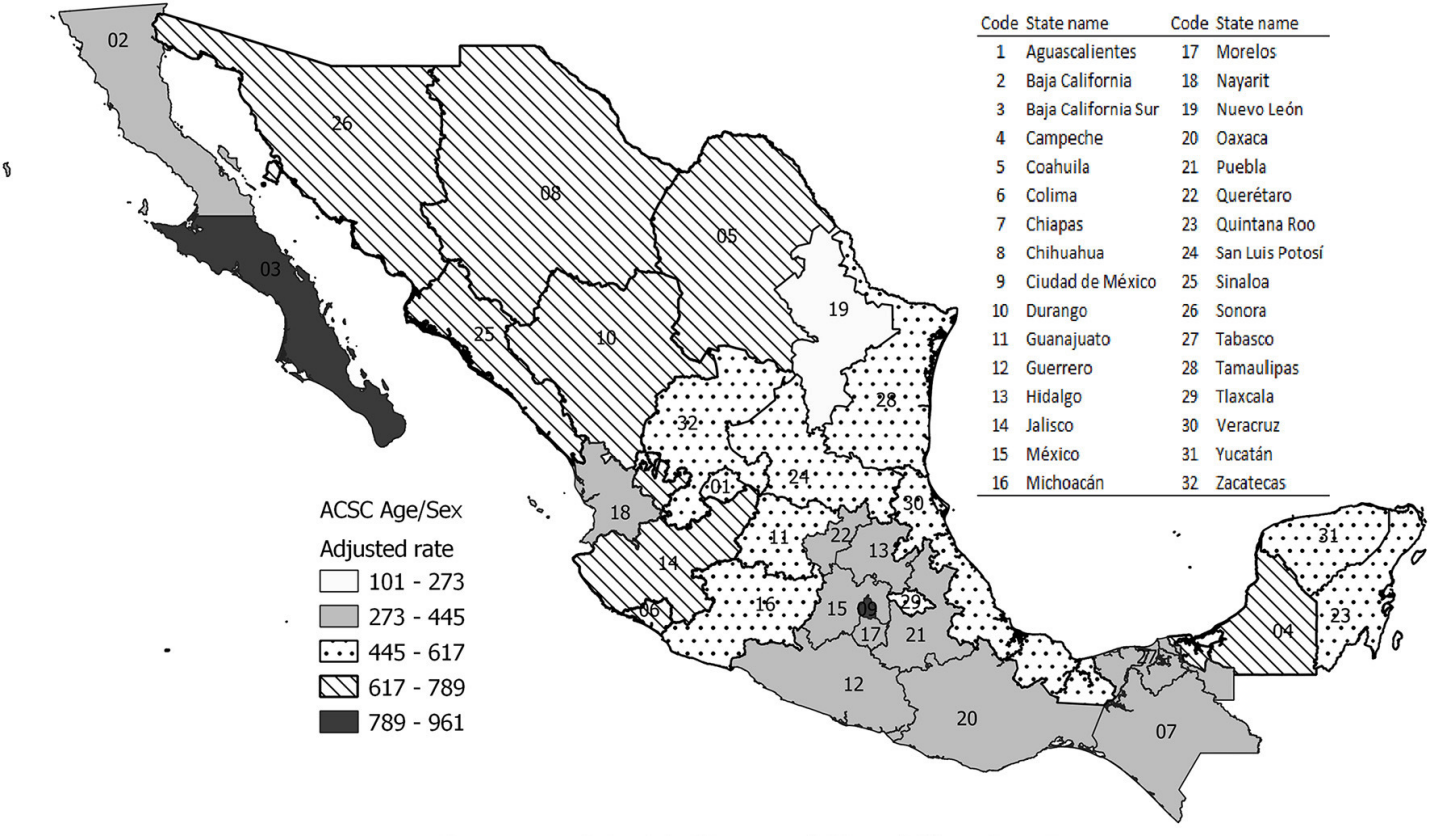
Geographic pattern of the AH rates by ACSC in México.

The rates of hospitalizations for ACSC adjusted by age and sex for each state in the country distributed in 5 groups from lowest to highest magnitude, as shown in Figure 1. The highest rates of avoidable hospitalizations were reported in Baja California Sur (960 per 100 thousand) and Mexico City (840 per 100 thousand), and the lowest rate was in the north of the country, in Nuevo León, with a rate of 101 per 100 thousand.

**Figure 2:**
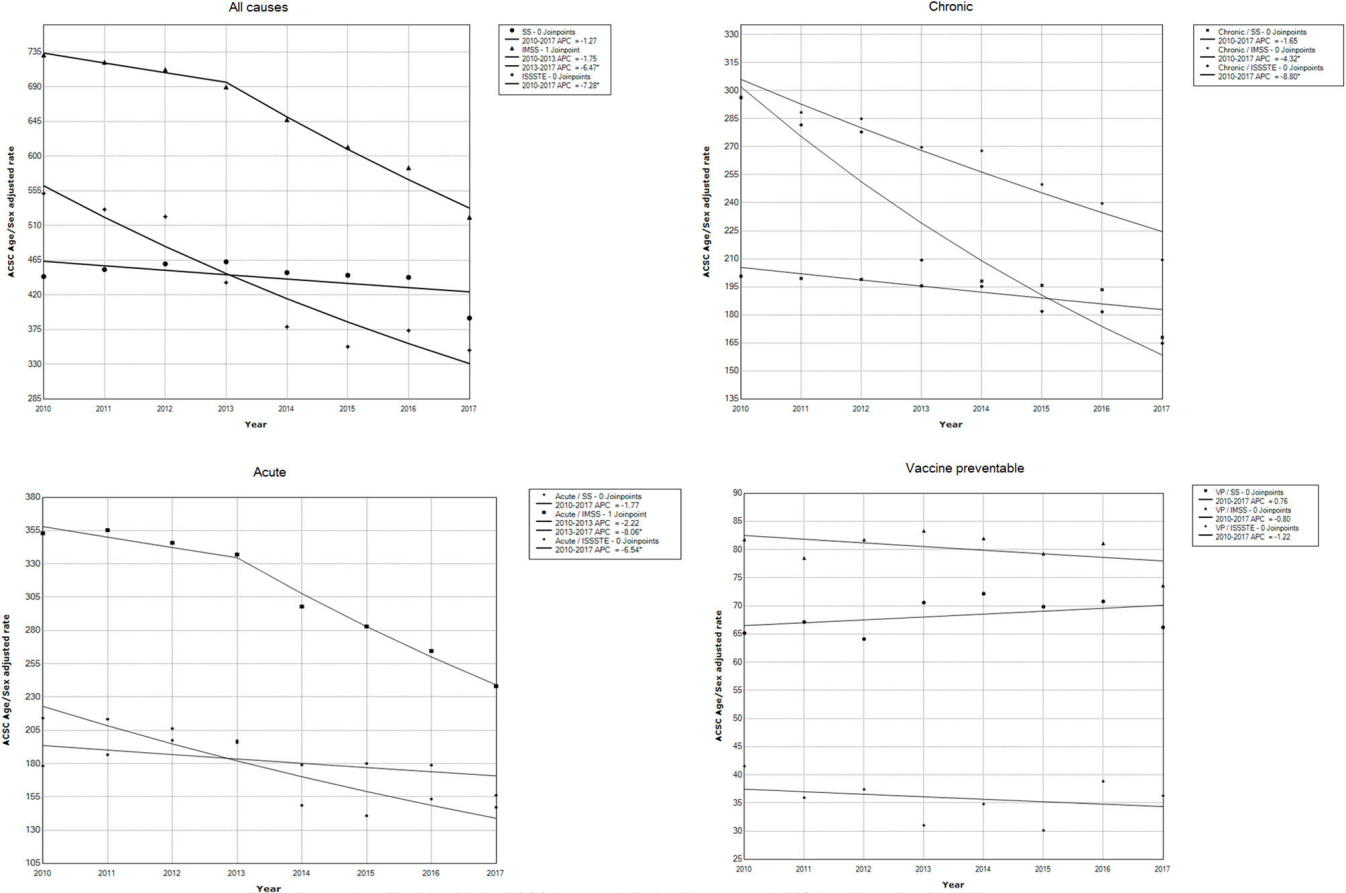
Join Point Regression Models. AH by ACSC in the public health system in México. Period 2010–2017.

The results of the joinpoint analysis by clinical condition categories for each institution, applied to the AH rates by ACSC for the years 2010–2017, are reported in Table 4. Figure 2 shows the trends according to the joinpoint identified in the analysis. For clinical conditions, in their different groups (all, vaccine-preventable, chronic and acute), an average annual decrease (AAPC) was observed for the period 2010–2017.

**Table 4:** Joinpoint analysis of age-sex standardized AH rates by ACSC categories by institution 2010–2017.

**Cause**	**Institution**	**Period**	**Change year**	APC (95% CI)^*^	**AAPC (95% CI)**
All causes	SS	2010–2017		−1.27 (−2.94, 0.43)	−1.27(−2.94, 0.43)
	IMSS	2010–2017			−4.47(−5.89, −3.04)
		2010–2013	2013	−1.75 (−5.81, 2.49)	
		2013–2017		−6.47 (−9.07, −3.79)	
	ISSSTE	2010–2017		−7.28 (−9.57, −4.92)	−7.28 (−9.57, −4.92)
Vaccine preventable	SS	2010–2017		0.76 (−0.86, 2.4)	0.76 (−0.86, 2.4)
	IMSS	2010–2017		−0.8 (−2.17, 0.58)	−0.8 (−2.17, 0.58)
	ISSSTE	2010–2017		−1.22 (−5.09, 2.81)	−1.22 (−5.09, 2.81)
Chronic	SS	2010–2017		−1.65 (−3.28, 0.01)	−1.65 (−3.28, 0.01)
	IMSS	2010–2017		−4.32 (−5.7, −2.93)	−4.32 (−5.7, −2.93)
	ISSSTE	2010–2017		−8.8 (−11.21, −6.32)	−8.8 (−11.21, −6.32)
Acute	SS	2010–2017		−1.77 (−4.14, 0.65)	−1.77 (−4.14, 0.65)
	IMSS	2010–2017			−5.6 (−6.86, −4.33)
		2010–2013	2013	−2.22 (−5.84, 1.53)	
		2013–2017		−8.06 (−10.36, −5.7)	
	ISSSTE	2010–2017		−6.54 (−9.45, −3.53)	−6.54 (−9.45, −3.53)

* *Only significant joinpoints (p < 0.05) are retained in the final model for each clinical condition*.

For all causes combined, for each institution, a downward trend curve is observed, although significant only for SS. The rates of standardized AH decreased significantly from 2010 to 2017. The average annual decreases for IMSS and ISSSTE were −4.47% [95% CI (−5.89, −3.04) and −7.28 (95% CI −9.57, −4.92], respectively. A joinpoint was detected in 2013 for the rates of AH in IMSS with annual change (APC) of −6.47% (95% CI −9.07, −3.79) (Table 4; Figure 2).

Vaccine-preventable conditions showed the most stable rates, although not significant. The rates of AH in SS increased <1%, but then remained stable, in contrast with IMSS and ISSSTE, whose trend curves were in reduction, with average annual change of −0.8% (95% CI −2.17, 0.58) and −1.22% (−5.09, 2.81), respectively.

Regarding the rates of standardized AH for acute conditions during the period 2010–2017, the slopes were constant toward reduction with statistically significant average annual change only for IMSS and ISSSTE. The only joinpoint detected in 2013 was for rates of hospitalization for ACSC in IMSS, with an annual change (APC) of −8.06% (95% CI −10.3, −5.7) (Table 4; Figure 2).

The adjusted rates for chronic conditions were toward reduction in the study period (2010–2017), with average annual change of −1.65%, −4.32%, and −8.8% in SS, IMSS, and ISSSTE, respectively.

## Discussion

As has been documented in other studies, the rates of avoidable hospitalizations for ambulatory-care sensitive condition are considered an indirect indicator of the quality at the primary care, as well as an indicator of access to health services (32). The results of this analysis show how the rates of AH for ACSC, while decreasing at the country level, have differential variations among the health institutions. These variations can be explained by the characteristics of the health care system, which have an impact on access to health services and the capacity of services to resolve the demand for care, which are differential.

In the Mexican health system, the primary care level is the gateway to the health system (33, 34). However, in the last 12 years the MoH has implemented programs to improve access to primary care, with interventions for certain acute and chronic conditions (35, 36). Nevertheless, some inequities in access are still observed, especially in areas where the geographic and cultural conditions make the use of health services difficult, as seen in the AH for ACSC by state (37).

Another aspect that must be considered is that Seguro Popular users do not have an income for the time it takes to go to a first level consultation, while social security users usually receive an incapacity and a payment for the day. This means that patients with acute and more complicated conditions go to the emergency department; although, unfortunately, we are unable to identify emergency admissions at this level. Besides that, the population may have to acquire or access to treatment in some cases, while in others, the difficulty may be due to the patient's needs and their perspective regarding the cosmovision about which is the most adequate handling, and family support, which comes closest to cultural and ideological factors, fundamental in their control, as in the case of chronic diseases such as DM.

However, the existing variability in the practice of health care professionals, with deficiencies in the processes of care, has been documented (38, 39). A hypothetical explanation refers to differences in the level of training of health professionals and the type of incentives in their performance. Without leaving aside what refers to the structure and resources of medical units, which in many cases are not sufficient or sustainable to face the health needs of the population.

On the other hand, although the results of the overall rate of AH for ACSC are low compared to those reported by other studies (40) according to the ICD 10 codes, in Mexico, there is variability among institutions and periods, as shown by our results. The rates of AH in IMSS, identified in previous studies, remained constant for the period 2001–2009, unlike that found in 2010–2017, where there was a reduction in AH for ACSC.

However, the most prevalent cause of AH is related with diabetes mellitus in both studies. It is possible that during the study period there was a strengthening of health care at the first level, as with the implementation of programs focused on improving the quality of care in chronic diseases, specifically in the population with diabetes mellitus. Another element to be considered is the operation of the network of services that seeks coordination actions from the first to second level in order to improve the quality of care.

When comparing the results with those of Lugo-Palacios (19) in the specific population of the SS, a great variability is seen among states, but the overall rate of AH in these results does not show changes, which could be due to the source of information and the type of analysis.

Heterogeneity was observed in the rates of AH among the subsystems and states. One explanation for this would be given by the differences in the population with regard to social, cultural and economic determinants that influence in the use of health services, in spite of the changes in the process in health care at the first level, as has been observed in other studies (35, 41). The differences observed in the AH for ACSC between men and women (data not shown on tables) may be explained by different patterns of morbidity and utilization of health services (42). Women use more first-level services than men, so this group would have less control of its health and would need more specialized services for complications at the second and third level of care (43). Men are more reluctant to seek health services, which affects the timeliness of care and increases the likelihood of a hospitalization that could have been avoided. There is no doubt that there is an impact on AH for ACSC of acute conditions by the jump observed in the year 2013 toward reduction in the rates; however, this is significant for the IMSS population.

One of the limitations of this study is that the information was obtained from three sources of information, SAEH, IMSS and ISSSTE, so there is a limitation in relation to the validity of the main and secondary diagnosis at the time of hospital discharge. Another limitation was the lack of other variables or relevant information that could explain the variations observed in the different states and among the public health institutions analyzed. It is considered important to identify other sources of information that would allow us to know the individual characteristics of the population that uses hospitalization services, as well as their trajectory through the health system, in addition to identifying the availability of first-level physicians, not only in public institutions, but also to review the participation of private institutions at the regional level. The limitation of using a code list to quantify the frequency and trend of AH for ACSC is that there is no strategy to identify those admissions that were transferred from another medical unit or hospital. In addition, it is important to consider the infrastructure of the hospital units, such as the number of beds, the number of professionals and the characteristics of the provision of services.

However, the available database used for the HA analysis does not have the information regarding the number of professionals, number of beds, to establish any associations.

The system of patients' transfers is not standardized and it is difficult to take it into account from a secondary source of information.

One of the challenges is to generate an integrated information system that will allow the registration of variables related to the infrastructure of the facilities, as well as human resources, and variables related to the process of care, communication and coordination with the network of services from the hospital to the primary care unit.

### AH for ACSC in the Context of COVID-19

The current activity of primary care health services has changed, as has hospital care. To ensure the response capacity of the health system, it has been necessary to expand the number of hospitals and ICU beds, but it has also been necessary to reorganize the path that patients must follow from the point of entry into the health system, at the different levels of care, especially in the most remote populations with little access to health services (44, 45). Many of the individuals who presented COVID-19 have chronic diseases whose control must be resolved at the first-level care. However, from the start of the pandemic, the organization of health services has restricted access to this group of patients due to their vulnerability and risk of contracting the SARS-COV2 virus, which can alter the clinical course of their health status. The results on the behavior of the COVID-19 disease show how the probability of presenting severe symptoms that require hospital management and ICU bed is greater in the population that presents diabetes mellitus, hypertension, obesity, and other chronic diseases (46). We consider it necessary to have pre-pandemic information, since during the year 2020 the quality of care could be affected by changes in surveillance and ambulatory control of people with chronic and other preventable diseases, which will possibly impact hospitalization and mortality rates.

## Conclusion

AH for ACSC can be used as proxy indicator of access to primary care, but it can also be a measure used to observe the availability of hospital services. It is observed that there are variations by institution. The variation observed in the CV and SCV among subsystems and states may be caused by inequities in the provision of services. It is necessary to analyze in more detail the structural factors of the services and the effect of COVID-19 on the burden of AH for ACSC in the Mexican health system.

## Data Availability Statement

The raw data supporting the conclusions of this article will be made available by the authors, without undue reservation.

## Ethics Statement

The studies involving human participants were reviewed and approved by Research and Ethics Committee of the National Institute of Public Health in Mexico. Written informed consent for participation was not required for this study in accordance with the national legislation and the institutional requirements.

## Author Contributions

OP and PS were responsible for the project. LT-A was responsible for writing and editing the paper. SF-H and AN were responsible for the analysis. All authors contributed to the article and approved the submitted version.

## Funding

Funding for this study was provided by the National Council of Science and Technology (CONACYT, FOINS 248938) to the Project Rates estimation and costs of the Avoidable Hospitalizations in México.

## Conflict of Interest

The authors declare that the research was conducted in the absence of any commercial or financial relationships that could be construed as a potential conflict of interest.

## Publisher's Note

All claims expressed in this article are solely those of the authors and do not necessarily represent those of their affiliated organizations, or those of the publisher, the editors and the reviewers. Any product that may be evaluated in this article, or claim that may be made by its manufacturer, is not guaranteed or endorsed by the publisher.
